# On the Role of the Head Ganglia in Posture and Walking in Insects

**DOI:** 10.3389/fphys.2020.00135

**Published:** 2020-02-21

**Authors:** Stav Emanuel, Maayan Kaiser, Hans-Joachim Pflueger, Frederic Libersat

**Affiliations:** ^1^Department of Life Sciences and Zlotowski Center for Neuroscience, Ben-Gurion University of the Negev, Beersheba, Israel; ^2^Fachbereich Biologie Chemie Pharmazie, Institut für Biologie, Neurobiologie, Freie Universität Berlin, Berlin, Germany

**Keywords:** walking, insect, cerebral ganglia, gnathal ganglia, motor control, central complex, posture, neuroethology

## Abstract

In insects, locomotion is the result of rhythm generating thoracic circuits and their modulation by sensory reflexes and by inputs from the two head ganglia, the cerebral and the gnathal ganglia (GNG), which act as higher order neuronal centers playing different functions in the initiation, goal-direction, and maintenance of movement. Current knowledge on the various roles of major neuropiles of the cerebral ganglia (CRG), such as mushroom bodies (MB) and the central complex (CX), in particular, are discussed as well as the role of the GNG. Thoracic and head ganglia circuitries are connected by ascending and descending neurons. While less is known about the ascending neurons, recent studies in large insects and *Drosophila* have begun to unravel the identity of descending neurons and their appropriate roles in posture and locomotion. Descending inputs from the head ganglia are most important in initiating and modulating thoracic central pattern generating circuitries to achieve goal directed locomotion. In addition, the review will also deal with some known monoaminergic descending neurons which affect the motor circuits involved in posture and locomotion. In conclusion, we will present a few issues that have, until today, been little explored. For example, how and which descending neurons are selected to engage a specific motor behavior and how feedback from thoracic circuitry modulate the head ganglia circuitries. The review will discuss results from large insects, mainly locusts, crickets, and stick insects but will mostly focus on cockroaches and the fruit fly, *Drosophila*.

## Introduction

**FIGURE 1:**
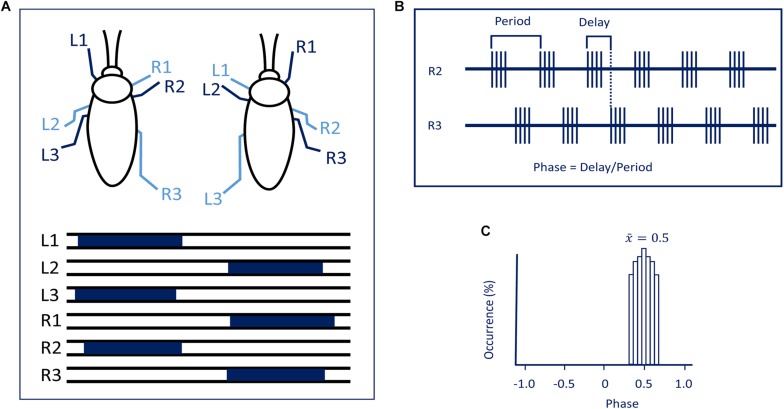
**(A)** The tripod gait predominantly used by cockroaches. Blue bars represent swing phases of the left (L) and right (R) front (1), middle (2), and hind legs (3) (modified with permission from Ayali et al., 2015). **(B)** To evaluate synchronization and coordination, activity (EMG) is recorded from the same muscle in two different legs. Period and time delay are measured cycle by cycle. **(C)** Relative occurrence of phases of R2 in the cycle of R3 is represented as a histogram. In the present case, the median phase is 0.5 showing that this pair of legs are active in anti-phase.

Most of us have heard of the expression “running around like a headless chicken.” Of course, headless chicken cannot walk or run in a coordinated manner and what is true about chickens is also true for insects. The neuronal control of rhythmic behaviors is regulated by two main functional circuitries that are similar in architecture in invertebrate and vertebrate systems. Such rhythmic behaviors have been examined in various groups of invertebrate phyla ranging from worms to mollusk to arthropods. A common feature in the organization of the neuronal circuit underlying such rhythmic behaviors is a task distribution along the animal’s nervous system axis. First, movements in a given appendage must be organized in a spatiotemporal pattern and be coordinated with other appendages (Figure 1). This function is generated by local networks generally referred to as CPGs. Such CPGs include motor and inter-neuronal constituents and are strongly modulated by peripheral sensory elements as well (Marder and Bucher, 2001; Marder et al., 2005; Selverston, 2010). Rhythmicity in these local networks is rarely expressed spontaneously (Knop et al., 2001) and often requires an appropriate mechanical or pharmacological stimulus (Arshavsky et al., 1997; Marder and Bucher, 2001; Knebel et al., 2019). In the absence of any sensory input, CPGs are able to generate a rhythm which pre-structures the rhythmicity but not the coordination pattern of the natural behavior (Knebel et al., 2017; review: Bidaye et al., 2017). However, if this rhythm exhibits similar phase relations as the natural behavior it has been called a fictive behavior (for example, fictive walking or stepping; Ryckebusch and Laurent, 1993). Typically, such functional unit namely the CPGs of a given locomotory behavior reside in the “lower” portion of the central nervous system: the ventral nerve cord. The second functional unit is that involved in initiation, modulation, maintenance, and termination of the rhythmic motor pattern generated in the nerve cord. These two functional units interact with feedforward and feedback interactions. While considerable knowledge has been gathered regarding the single neuron and circuit architecture of CPGs and the interactions between sensory and central components of such circuits including an arsenal of reflexes confined to the thoracic ganglia (Burrows, 1996), less is known regarding the role of head ganglia in the control of such circuitries.

As far as head ganglia are concerned, a difference exists between hemimetabolous (stick insects, locusts, cockroaches) and holometabolous insects (fruit flies). In hemimetabolous insects, the GNG (formerly known as the “subesophageal ganglion”), ventral to the gut, is well separated by CirC from the CRG (formerly known as “supraesophageal ganglion” or brain). In holometabolous insects, the GNG are more or less fused with the CRG to form one cerebral mass with the gut running through an opening in between (the GNG are only indicated by being located ventral to the gut in this otherwise uniform cerebral structure) and no CirC are visible from outside. To understand the interactions between the thoracic circuits and those in the head ganglia, various rhythmic behaviors have extensively studied. While several rhythmic behaviors in insects can be executed without the head ganglia meaning both the cerebral and GNG, for example, flight (Wilson, 1961; Gal and Libersat, 2006) righting (Gal and Libersat, 2006), and grooming with the metathoracic legs (Eaton and Farley, 1969; Reingold and Camhi, 1977; Berkowitz and Laurent, 1996), others such as walking will be poorly or not performed in the absence of the head ganglia. The role of the head ganglia in the orchestration of walking has been addressed in several reviews (Heinrich, 2002; Borgmann and Bueschges, 2015; Bidaye et al., 2017) and there is task specificity between CRG and GNG. That being noted, there has been considerable progress unraveling the action of the descending control from the CRG of insects taking advantage of neurogenetic tools in *Drosophila* and a combination of behavioral analysis and electrophysiology in large insects. A review covering at any depth all circuit organization that generates behaviors as diverse as those mentioned above would be unmanageable. Hence, in this review, we will limit our focus on the role of the head ganglia in posture and walking in insects. Until quite recently the bulk of work on this topic has been done on large hemimetabolous insects such as locusts, stick insects, crickets, and cockroaches. But today, the fruit fly, *Drosophila*, has also become a major model for investigating the neuronal basis of walking from circuits to behavior. In the fly, one can use mutations of discrete areas of the central nervous system as genetic tools to identify specific neurons and networks and manipulate or monitor their activity. But with this mind, one should not forget that *Drosophila* stands on the shoulders of giants. Among those giants, we will focus our review on cockroaches which have been extensively studied with regard to walking (Ritzmann and Zill, 2017). Moreover, as most studies have been carried out on the adult stage of large insects, we limit our comparative and complementary survey on walking in the adult *Drosophila*. Insects belong to the subphylum *Hexapoda*, characterized by three pairs of legs. Most insects use, at some speed, the alternating tripod ground locomotion in which three legs are on the ground when the other three are off the ground. Adjacent legs from the same segment are in antiphase while legs from two consecutive segments on opposite side of the body move nearly synchronously (Figure 1). Other coordination patterns do occur speed-dependently as well (Wosnitza et al., 2013).

## The Cerebral Ganglia (CRG)

**FIGURE 2:**
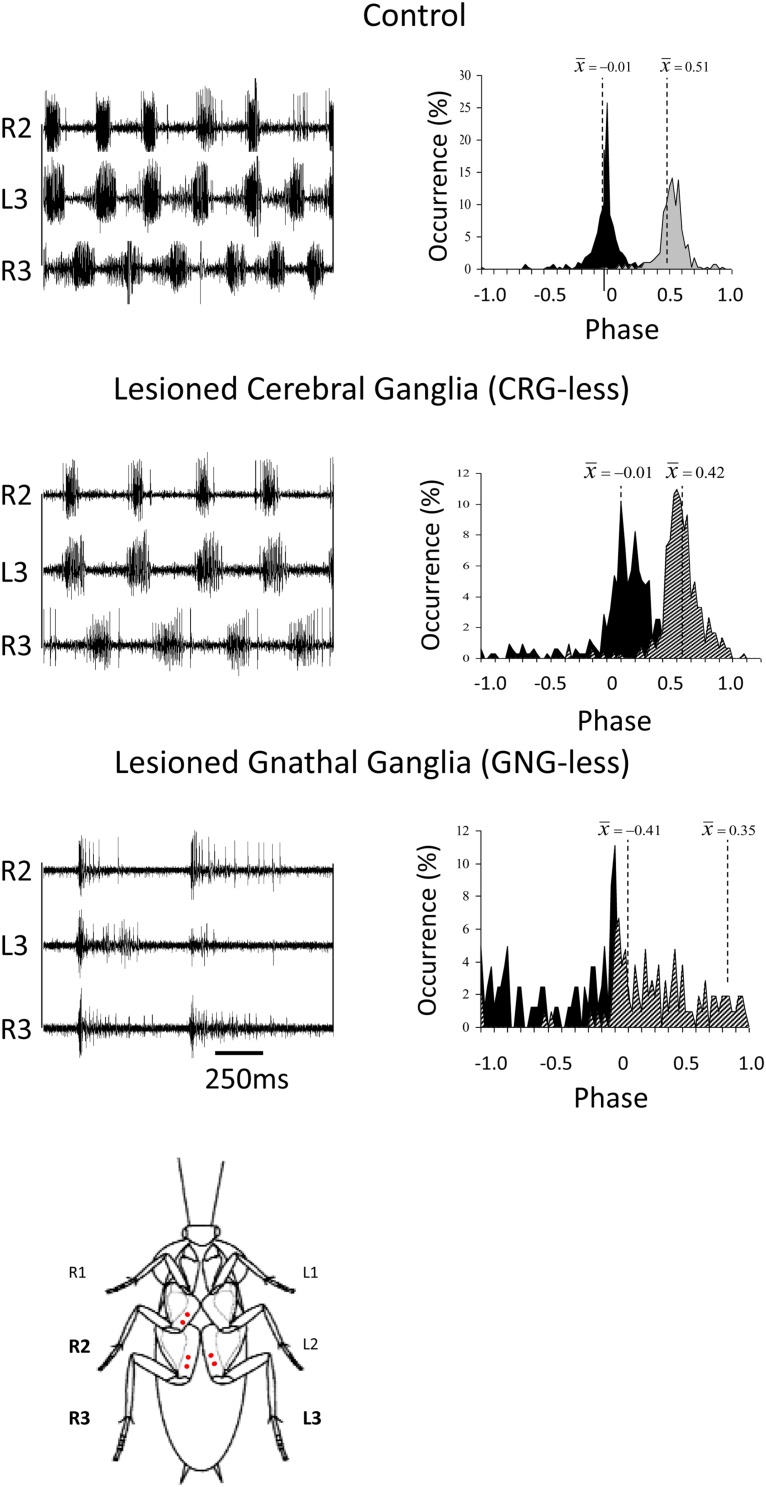
Coordination in cockroaches with head ganglia lesions. Left panels: EMG traces from the coxal depressor muscles, demonstrating the coordination between the right mesothoracic leg (R2) and the right and left metathoracic legs (R3 and L3) in control, CRG-less, and GNG-less cockroaches. Spontaneously initiated locomotion of control and brainless cockroaches is characterized by the tripod-gait coordination. GNG-less cockroaches fail to show spontaneous tripod-gait coordinated locomotion. Right panels: Relative occurrence of phases of R2 and R3 in the cycle of L3. The median phases are also indicated. Note the lack of tripod-gait coordination in GNG-less cockroaches (adapted with permission from Gal and Libersat, 2006). A cockroach is also shown ventral side up with red dots indicating the location of the insertion of the EMG electrodes.

The CRG (formerly brain or supraesophageal ganglion) and GNG (formerly subesophageal ganglion), which are connected to each other by the CirC in large insects, are considered as “higher order” neuronal centers which modulate different aspects of locomotion (Figure 2). The picture that emerges from early and recent research is that the CRG are primarily but not exclusively involved in initiation, regulation, and probably termination of walking. The GNG, in contrast, are predominantly involved in coordination as we shall discuss later.

**FIGURE 3:**
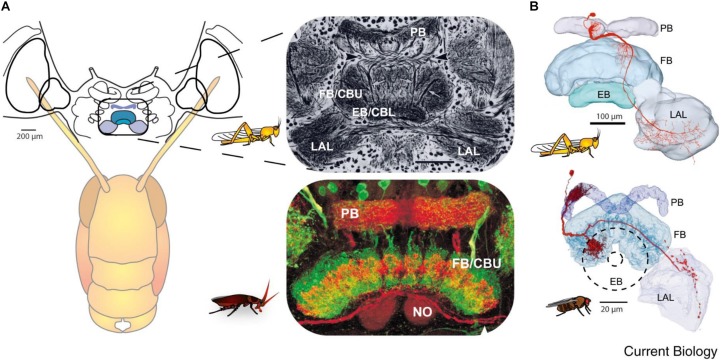
Similarity of the anatomy of the central complex across species. **(A)** Overview of the central complex and support structures: The protocerebral bridge (PB), fan-shaped body (FB) or central body upper (CBU), ellipsoid body (EB) or central body lower (CBL), noduli (NO), and lateral accessory lobe (LAL) in the locust and cockroach. **(B)** Same like in **A** for locust and fly (reprinted with permission from Turner-Evans and Jayaraman, 2016; with permission of Copyright Clearance center, license number 4597641450839 and courtesy of Vivek Jayaraman).

Insects with the CirC cut exhibit long bouts of unoriented walking activity (Roeder, 1937; Bässler and Wegner, 1983; Kien, 1983; Ridgel and Ritzmann, 2005) suggesting that the CRG is a source of inhibitory influence on thoracic walking centers. CRG removal in stick insect (*Carausius morosus*) or cockroaches (*Periplaneta americana*) has little effect on tripod-gait coordination in walking, though it mildly increases the variability in inter-leg phase relationship (Figure 2). The latter suggests that the “fine tuning” of the inter-leg coordination might be controlled by the CRG which integrates visual, olfactory, and tactile-antennal information (Graham, 1979; Gal and Libersat, 2006). In contrast, lesions of the neck connectives, those between the GNG and the first (pro)thoracic ganglion, dramatically decrease spontaneous and evoked walking in locusts (Kien, 1983). Gal and Libersat (2006) demonstrated the inhibitory neural influences on walking behavior by examining cockroaches following removal of the CRG. Using more discrete lesions of the CRG in praying mantis, Roeder (1937) was able to identify that the locomotor inhibiting center lies in the dorsal region of the protocerebral ganglion and suggested it is the MB. It is a few decades later that Huber (1960) showed that stimulation of the MB usually inhibited walking in crickets whereas stimulation of another CRG structure, the CX, initiated walking. In support of this, Martin et al. (1998) showed that MB suppress locomotor activity in *Drosophila melanogaster*. Electrophysiological recordings from cockroach MB neurons in freely moving cockroaches revealed several classes of neurons associated with movements (Mizunami et al., 1998a,b,c). Moreover, motor behavioral measurements over period of weeks of MB defective flies showed that MB suppress activity (Helfrich-Förster et al., 2002). In cockroaches, procaine (a reversible voltage dependent sodium channels blocker) injection to the MB increases spontaneous walking (Kaiser and Libersat, 2015). In *Drosophila*, the MB are also required for daily rhythmic locomotor activity (Mabuchi et al., 2016) and enhance motor activity in the beginning of light-evoked walking (Serway et al., 2009). Hence, all aforementioned experimental evidence suggests that the MB have a regulatory role for walking related locomotion. When looking for a CRG structure that is permissive on walking, the CX was shown to be critical in the selection of motor actions. The CX exhibits an elaborate CRG center in its layered architecture. The CX is defined as a group of four midline neuropils: the PB, the FB or (central body upper: CBU), the EB (or central body lower: CBL), the NO, and LAL (Figure 3). Research across many species has shown that the CX contains the circuitry for elementary navigational decisions (for a recent review: Honkanen et al., 2019). Its function as a multi-sensory processing hub has been reviewed elsewhere (Pfeiffer and Homberg, 2014; Homberg, 2015). While numerous investigations have shown that the CX is involved in sensory integration and pre-motor processing, others have uncovered its role in the initiation and ongoing regulation of locomotion. We will first discuss the role of the CX in the initiation and regulation of walking and posture.

### The Central Complex (CX) and the Initiation and Maintenance of Walking

**FIGURE 4:**
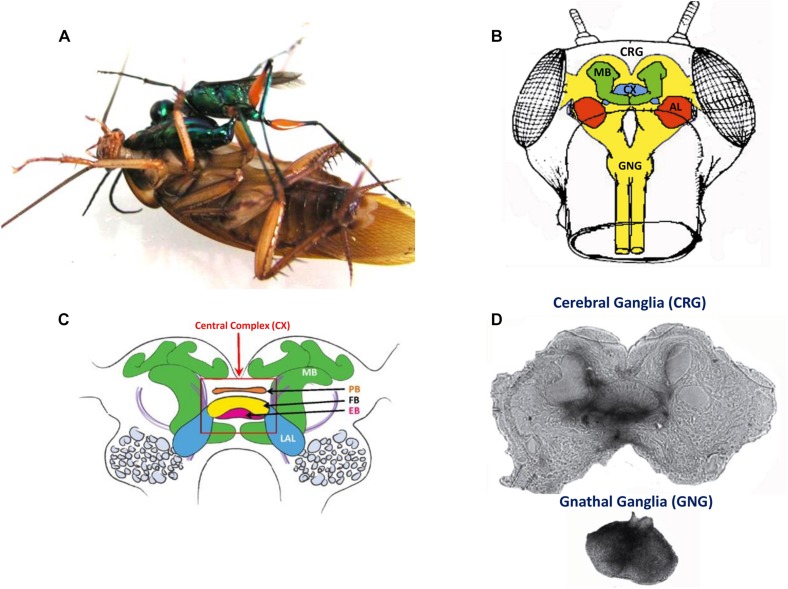
**(A)** An adult wasp stings a cockroach into the head to manipulate the cockroach behavior. **(B)** Drawing of a frontal view of the cockroach head ganglia (CRG: cerebral ganglia, CX: central complex, AL: antennal lobes, MB: mushroom bodies; GNG: gnathal ganglia). **(C)** Diagram of the cockroach CRG. Central complex is within red rectangle. PB: protocerebral bridge, FB: fan-shaped body, EB: ellipsoid body, LAL: lateral accessory lobes, MB: mushroom bodies (reproduced with permission from Ritzmann et al., 2012; courtesy of Josh Martin and Roy Ritzmann; License: CC BY 4) **(D)** Autoradiographs of the cerebral ganglia and the gnathal ganglia of a cockroach stung by a radio-labeled wasp. Black staining indicates the presence of venom (adapted with permission from Haspel et al., 2003).

Kaiser and Libersat (2015) showed that injection of procaine to the CX results in a decrease in spontaneous walking. *Drosophila* CX mutants, with altered internal CX neuropils (the PB, the FB, and the EB), show a decrease in walking activity. Walking speed and step length as a function of the stepping period are both reduced (Strauss and Heisenberg, 1993). While locomotor activity in these mutants is clustered in bouts that are initiated at a normal frequency, their duration is reduced and the interval between bouts is increased (Martin et al., 1999). However, these studies did not find deficits in the initiation of the locomotor activity. But in other studies, some of the strains of *Drosophila* CX mutants are less likely to initiate walking (Heisenberg, 1994; Strauss, 2002). To further investigate the possible role of the CX in walking, Bender et al. (2010) achieved chronic tetrode recordings from the CX of tethered but otherwise intact cockroaches performing stationary walking or walking on a slippery surface. The recorded neural activity was correlated with stepping rate. Moreover, electrical stimulation of the CX could initiate or regulate walking (Bender et al., 2010). Recent advances in understanding the mechanisms by which the initiation and maintenance of walking in cockroaches come from an unexpected source of natural history: the zombification of cockroaches by a parasitoid wasp (Figure 4). The American cockroach (*P. americana*) can fall victim to the parasitoid Jewel Wasp (*Ampulex compressa*), which uses them as live and immobile food supply for its larva. This wasp stings directly inside the cerebral and GNG in the head capsule to immobilize its cockroach prey. The venom injected in the head ganglia induces a dramatic change in the cockroach’s ability to show spontaneous walking and to escape from tactile or wind stimuli. While unresponsive, the wasp cuts off both antennae to feed on the cockroach hemolymph from the broken ends. The wasp then grasps one of the antennal stumps and, walking backward, leads its prey to a pre-selected burrow to be later consumed by a single wasp larva. The highest concentration of venom was localized in and around the CX (Haspel et al., 2003).

Using affinity chromatography and Label Free Quantitative Mass Spectrometry (LFQMS), Kaiser et al. (2019) found that the venom binds to synaptic proteins and that numerous proteins are differentially expressed in the CX of stung cockroaches. Many of differentially expressed proteins are involved in signal transduction pathways, such as the Rho GTPase pathway, which is implicated in synaptic plasticity. This suggests that the Jewel Wasp exerts control over cockroach behavior through a molecular cross-talk between venom components and primarily neuronal molecular targets in the host CRG, leading to broad-based alteration of synaptic efficacy in the CX. Such a decrease in synaptic drive to the CX would result in a decrease in descending activity and a reduction in excitatory drive from the CRG to the thoracic motor circuitries. This may account for the observed motor impairments induced by the venom. In support of this, removing the input from the CRG (or “brain”) or after a wasp sting, the activity of thoracic octopaminergic neurons is altered (decreases) in stung and “brainless” animals (Rosenberg et al., 2006). The alteration in the activity of octopamine neurons may be part of the mechanism by which the wasp induces a change in the behavioral state of its prey.

Distinct peptidergic pathways in the CX have specific roles in the fine tuning of locomotor activity of *Drosophila.* Two different neuropeptides in *Drosophila’s* CX, Drosophila tachykinin (DTK) and short neuropeptide F (sNPF), are shown to modulate locomotor activity (Kahsai et al., 2010). DTK is expressed in two specific populations of neurons innervating the FB and in other CX neurons. RNA interference (RNAi) directed to this peptide caused increasing avoidance behavior and increased number of activity–rest bouts. sNPF is expressed in CX neurons and inhibits the overall motor activity level, as reduced sNPF levels by RNAi in those neurons increased distance traveled and mean walking speed (Kahsai et al., 2010).

Walking behavior is strongly related to rest-activity cycle (sleep cycle) of the animal. Therefore, walking behavior must be under the control of a sleep control system. In *Drosophila*, a circuitry for inducing sleep is well established and involves CX neuropils (Donlea et al., 2011, 2014; Liu et al., 2016; Pimentel et al., 2016). It was recently shown by Donlea et al. (2018) that dFB neurons induce sleep by inhibitory transmitters including neuropeptide allatostatin-A (AstA). Among the targets of AstA is a group of interneurons in the EB of the CX that they named “helicon cells.” These neurons are inhibited by sleep-promoting AstA, excited by visual input, and are permissive for locomotion. Therefore, one way of inducing a rest period is by inhibition of walking-permissive circuits in the *Drosophila* CRG. Another way to modulate walking behavior is in the activity of CRG circuits that promote wakefulness. In *Drosophila*, two dopaminergic neurons signal to the dFB and their activity reduces sleep and promotes arousal (Liu et al., 2012).

### The Central Complex (CX) and Navigation in Walking

After a mid-line section and removal of one side of the CRG, a mantis makes small circular walking toward the intact side. These circular movements are due in part to the unequal right–left body tonus (Roeder, 1937). Research across many species has shown that the CX contains the circuitry that process sensory information to perform proper navigation (Honkanen et al., 2019).

Analysis of recording of the CX in freely moving cockroaches leads to the grouping of movement-predictive cells for slow or fast forward walking, left or right turns, or combinations of forward and turning speeds (Martin et al., 2015). Moreover, they showed that the CX via its descending output neurons may modulate leg reflexes in the thorax to facilitate turning. The role of certain sub-regions of the CX is uncovered by cockroaches with lesions to the PB and EB that exhibit turning deficits (Harley and Ritzmann, 2010). Moreover, stimulation through recording wires inserted in the CX produced consistent trajectories of forward walking or turning in these animals (Guo and Ritzmann, 2013). They concluded that asymmetrical activity in the CX precedes and influences cockroach turning behavior. More recently, Varga and Ritzmann (2016) uncovered head-direction cells in the CX of the cockroach. Such cells encode the animal’s heading relative to a landmark’s position in several ways. Some cells are tuned to a particular direction apparently rely on internal cues while others rely on external sensory cues (Varga and Ritzmann, 2016).

It was recently shown that *Drosophila* follow straight courses relative to landmarks (Green et al., 2018). Wolff et al. (2015) had recently mapped the structure of the PB of the *Drosophila* CX and identified several classes of neurons. A class of columnar neurons of the PB which sends dendrites to a specific area of the EB encodes the fly’s azimuth relative to its environment. These neurons (termed EB-PB-gall or E-PG neurons) were shown to be tracking the fly’s angular movements (heading) even in darkness (Seelig and Jayaraman, 2015; Green et al., 2017; Turner-Evans et al., 2017). Inhibiting synaptic transmission in E-PG neurons destabilized these neurons phase activity and reduced the distance and speed of walking (Green et al., 2018). These findings, however, do not fully account for the body angle tracking necessary for directional navigation over distances.

### The Central Complex (CX) and Posture

**FIGURE 5:**
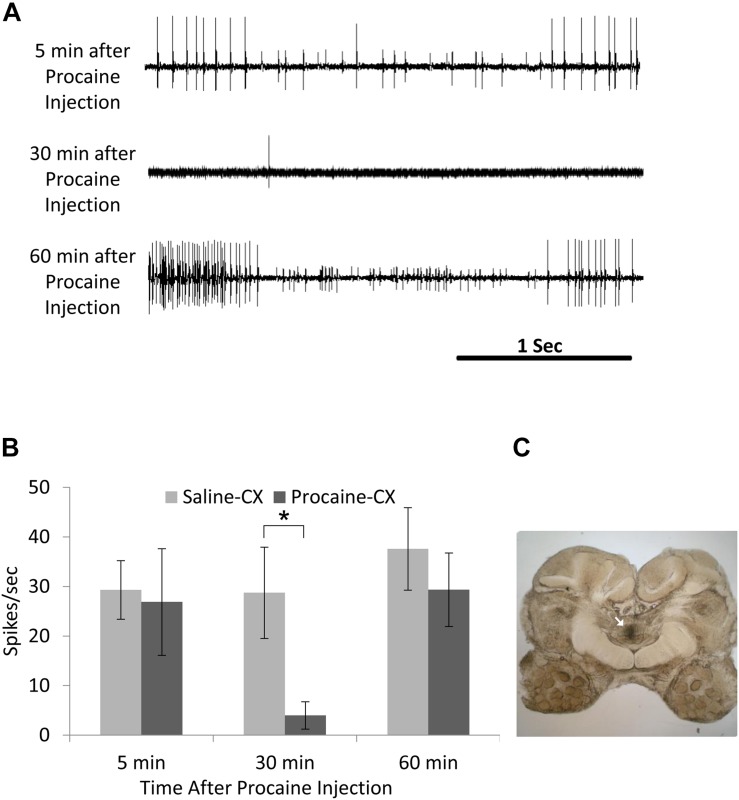
Activity in coxal slow motoneuron recorded as EMG spikes from the coxal depressor muscle after procaine injection to the central complex (CX). **(A)** Representative EMG recordings traces of coxal slow motoneuron ongoing activity 5 min after (upper trace; before procaine effect), 30 min after (middle trace; peak procaine effect), and 30 min after (lower trace; recovery from procaine effect) procaine injection to the CX. **(B)** Comparison of EMG spikes (spikes/sec) following saline (control; *n* = 6) and procaine (*n* = 6) injection to the CX. A significant decrease (*P* < 0.05; *t*-test) was found 30 min after procaine injection as compared to 30 min after saline injection. Each bar represents the averaged spikes/second ± SEM; significance is indicated with asterisk. **(C)** A representative image for postmortem verification of injection site. Arrow indicates the injection site in the fan-shaped body of the CX (adapted with permission from Emanuel and Libersat, 2017).

First, it should be noted that posture is also handled at the single ganglion level by feedback from sense organs, primarily campaniform sensilla embedded in the cuticle of the legs (Zill et al., 2004). But reflexes handled at the local level of thoracic ganglia can be modulated by the CX. Hence, electrical stimulation of the CX in a restrained animal while recording from the slow depressor motoneuron (Ds) impacts on the femoral chordotonal organ to Ds reflex pathway (Martin et al., 2015). In praying mantis, removal of the CRG results in great decrease of tonus on both sides of the body (Roeder, 1937). Likewise, normal posture is altered in headless cockroaches (Ridgel and Ritzmann, 2005). But immediately after lesion of the neck connectives, cockroaches first show a hyper-extended posture, which is decreased thereafter over a few days. In contrast, the posture of cockroaches with lesions of the CirC appears similar to intact cockroaches. Yet, when challenged with the more difficult task of climbing, cockroaches with their CirC cut show a postural deficit (Ritzmann and Quinn, 2004) which affects their ability to climb up on smooth inclines. When confronted with a substantial incline, these cockroaches fail systematically to manage this obstacle. The ongoing activity of the coxal slow motoneuron ongoing activity, known to be involved in posture, is reduced after injection of procaine into the cockroach’s CX (Emanuel and Libersat, 2017). Moreover, the regular tonic firing of the slow motoneuron is greatly reduced in cockroaches stung by the jewel wasp (Emanuel and Libersat, 2017). Hence, postural tonus might be mediated by the head ganglia pre-motor circuits since injection of procaine to the CX decreases coxal slow motoneuron activity in a similar manner to the wasp venom injection in the CX (Figure 5).

### The Central Complex (CX) Connectivity to Descending Interneurons (DINs)

#### Neuroanatomy of the DINs

The sensory information processed in the CX must be transferred to DINs projecting to the ventral nerve cord. There are approximately 200 pairs of neurons in the CRG that have axons that descend to and beyond the thoracic ganglia in cockroaches (Okada et al., 2003) and crickets (Staudacher, 1998). In addition, there are another 100 neurons that connect the CRG only with the GNG in locusts (Heinrich, 2002). In cockroaches, the dendrites of such DINs are distributed in most CRG areas, including the MB and the CX (Okada et al., 2003), two structures that have been implicated with walking. In crickets, none of the DINs identified so far extend collaterals into the CX and the MB. This difference could be due to incomplete staining of the small dendritic branches of DINs in crickets. Neurons leaving the CX project to a region of the CX termed the LAL, and several neurons that ultimately descend to the thoracic ganglia also pass through this region. Although the anatomical studies in cricket (Staudacher, 1998) and cockroach (Okada et al., 2003) have identified how DINs are organized, the lack of genetic tools in these insects currently limits a systematic investigation of their roles in motor control. A comparison of a recent study on *Drosophila* DINs shows a high degree of conservation in the number and organization of DINs in both hemimatebolous and holometabolous insects (Hsu and Bhandawat, 2016; Namiki et al., 2018). There are 350 pairs of DINs in the fly head ganglia of which roughly 180 pairs are located in GNG (Namiki et al., 2018). These numbers compare well with those reported in crickets and cockroaches and the spatial distribution of DINs in the CRG in clusters is also similar in all three species (Hsu and Bhandawat, 2016). Likewise, the number of DINs in *Drosophila* GNG is 121 pairs and comparable to the roughly 150 pairs reported in locusts (Knebel et al., 2019). Interestingly, DINs of cockroaches and flies receive input from regions of the CRG that are innervated by outputs from MB and CX (Hsu and Bhandawat, 2016). In *Drosophila*, none of the DINs originate in the CX or MB (Namiki et al., 2018). Equally importantly, none of DINs innervate the CX or MB, implying that these neuropils do not directly affect motor output. Roughly 65% of the DINs send an axon in neck connective ipsilateral to their soma (Namiki et al., 2018).

#### DINs and Walking

In an attempt to record and identify DINs, Böhm and Schildberger (1992) found neurons that changed their levels of activity when the animal was walking. The activity of two DINs was directly correlated with walking activity and seemed to control walking parameters. Activation of one DIN caused the resting animal to begin walking, and interrupting the discharge of this DIN brought the walking animal to a halt. While investigating DINs associated with phonotaxis in crickets, Zorović and Hedwig (2013) recorded intracellularly at least four DINs while the animals were standing or walking on an open-loop trackball system. They found that activity of all four DINs was correlated to forward walking. Furthermore, injection of depolarizing current elicited walking and/or steering in three of four neurons. Some neurons showed arborizations in the LALs supporting the hypothesis that this region bridges between the CX and the DINs. Activation of different DINs in *Drosophila* induces running or freezing in a state-dependent manner (Zacarias et al., 2018). Some DINs when stimulated induce backward walking in *Drosophila*. The DIN which earned the name of moonwalker descending neuron (MDN) is required for flies to walk backward and is sufficient to trigger backward walking under conditions in which flies would otherwise walk forward (Bidaye et al., 2014). Optogenetic activation of 26 DINs drove locomotion behaviors such as slow locomotion, fast locomotion, and global increase in locomotor activity (Cande et al., 2018). These activated behaviors were often dependent on the behavior of the fly immediately before descending neuron activation, indicating a context dependent role for these descending neurons.

## The Gnathal Ganglia (GNG)

In contrast to headless insects, animals in which the CRG have been removed (or the CirC severed) engage longer bouts of spontaneous walking than intact animals. There are about 90 DINs with a cell body in the GNG most of which have a contralateral axon (Kien et al., 1990; Gal and Libersat, 2006). Such a number might be an underestimate as a recent study labeled roughly 150 descending GNG neurons (Knebel et al., 2019). In *Drosophila*, the GNG DINs group provides the major pathway to the leg motor neuropiles in the ventral nerve cord (Namiki et al., 2018). Locust GNG DINs show an elevated activity during and after the preparatory phase of walking (Kien, 1990a). These neurons typically fire throughout the walking bout, as temporally structured patterns that are not directly correlated with the stepping cycles (Kien, 1990b). Performing a mid-sagittal section through the GNG removes the effect of GNG DINs on thoracic CPGs while leaving the connectivity between the CRG and thoracic ganglia (the “through running axons” of CRG DINs, B-DINs) (Gal and Libersat, 2006). The direct descending pathway from the CRG to the thorax (the “through running axons”) is mostly unaffected by this midline cut since this pathway runs through the margins of the GNG (Altman and Kien, 1987). Such operated animals show very little, if any spontaneous walking. But if challenged to “walk” on water, their leg movements are mostly uncoordinated (Gal and Libersat, 2006). This is in sharp contrast to the walking pattern of cockroaches after removal of the CRG which show normal coordination both on land and water. This strongly suggests that the GNG is involved in leg coordination.

## Monoaminergic Descending Interneurons (DINs) and Descending Control of Walking

Multiple neurotransmitters have been found to be manufactured by the DINs. These include acetylcholine, GABA, glutamate, serotonin, dopamine, and octopamine (Hsu and Bhandawat, 2016). Hsu and Bhandawat (2016) also found that acetylcholine and GABA, the major excitatory and inhibitory neurotransmitter, respectively, are produced equally and represent roughly 75% of the DINs population (Hsu and Bhandawat, 2016). The monoamines serotonin, dopamine, and octopamine are known to modulate well-defined behaviors. The idea of chemical coding of specific behaviors was born out of experiments showing that local application of certain monoamines in the central nervous system can reproducibly evoke a single coherent behavior (Libersat and Pflueger, 2004). This idea was coined as the orchestration hypothesis by Graham Hoyle when he and his colleagues revealed that injection of octopamine in the locust thoracic ganglia released flight-like behavior (Sombati and Hoyle, 1984). This observation was later confirmed in *Drosophila* where application of these compounds induces a set of complex behavioral responses in the decapitated flies including the stimulation of grooming and locomotion (Yellman et al., 1997). In fact, octopamine injected into the neuropiles of locust thoracic ganglia primes the motor system to flight in contrast to other modulators such as pilocarpine or tyramine that act concentration dependently and can release either fictive stepping/walking or fictive flight or both simultaneously (Rillich et al., 2013). One population of octopaminergic DINs, with unpaired median morphology, originates in the GNG of locusts (Bräunig and Burrows, 2004). Recently, a cluster of DINs of the deutocerebrum containing tyramine/octopamine has been identified in cockroaches, stick insects, and locust (Kononenko et al., 2019). In locusts, these DINs synthesize octopamine from tyramine only after the insect experiences stimuli associated with stress and, thus, these DINS could convey information about the “stress status” to thoracic circuits. It would be interesting to identify such octopaminergic DINs in *Drosophila* head ganglia and manipulate their activity in the intact adult to observe their impact on walking.

Taking advantage of the neurogenetics tools available with *Drosophila* model system, Tschida and Bhandawat (2015) found two dopaminergic DINs in *Drosophila* GNG and tested whether their activity promotes walking. This investigation was motivated by previous studies in reduced preparations which showed that application of dopamine receptor agonists elicits fictive motor rhythms. The authors found that dopaminergic DINs activity correlated with certain leg movements and walking speed. But increasing dopaminergic DINs activity failed to impact on walking (Tschida and Bhandawat, 2015).

## Conclusion and Future Prospects

While numerous investigations have shown that the CRG, and in particular the CX is involved in sensory integration and processing, for example, of polarized light, others have uncovered its role in the initiation and ongoing regulation of locomotion. First, the CX appears to be involved in the arousal state of insects in preparation for the initiation of walking. Further experimental evidence indicates its role in the initiation and maintenance of walking. Asymmetrical recruitment of the CX also reveals its involvement in turning and negotiating obstacle during ongoing walking. How is the CX premotor command transferred to DINs is not known but some experimental evidence points to a region of the CX called “the LAL” (Namiki and Kanzaki, 2016). The GNG, also part of the head ganglia, and origin of many DINs appear to be involved in maintenance and inter-leg coordination of walking. Numerous DINs organized as clusters project downstream to motor centers in the nerve cord. How these neurons are involved in the initiation and regulation of coordination for steering is still unknown. Likewise, the connectivity between the CX and the DINs needs attention. Therefore, the studies compiled and reviewed here raise a number of questions where insects may still have to offer interesting insights in the neural basis of locomotion.

For instance, all moving animals require that stimuli generated by self-motion be discarded and information on self-generated movements have to be integrated into feedback systems. Therefore, circuits of corollary discharge or efference copies are most likely a component of all sensory-to-motor systems in insect and vertebrate respectively (Poulet and Hedwig, 2006; Combes et al., 2008). In contrast to most vertebrates, most insects do not possess eyes that can be moved separately from the head and, thus, for gaze stabilization the whole head has to be moved, mainly by the neck muscles which means one degree of freedom less. However, many insects and cockroaches in particular, possess long movable antennae which act as active sensors for olfactory and tactile (“haptic”) stimuli. They are moved by muscles inserting at the base of the antenna. How neck and antennal muscles are activated during walking is, to our knowledge, less studied.

The nature of the feedforward and feedback loops between the cerebral and the GNG and how these are involved in the control of walking is largely unexplored. A further unresolved issue in insects is the nature of the thoracic ascending input to the head ganglia and what is its role in fine tuning walking initiation, regulation, and coordination. Bidaye et al. (2014) addressed this issue by studying descending control of backward walking in flies. Briefly, they identified a descending neuron that initiate backward walking (MDN) and an ascending neuron (MAN: moonwalker ascending neuron that appears to inhibit forward and facilitate backward walking. Finally, there must be location in the CRG where the sensory information from the head sensors (goal direction) and legs sensors (proprioception) converge for appropriate initiation and coordination of walking.

The expression of multiple neuropeptides and neurotransmitters in *Drosophila* and other insect CX must be associated with different functional roles. As several neuropeptides are expressed in the circuitry of the CX, some may be modulators of locomotor behavior. The CX complex is rich in neuroactive substances. Among the biogenic amines and neuropeptides substances in neurons or innervating neurons of the CX are octopamine, serotonin, dopamine and allatotropin, leucokinin, myoinihibitory peptide, RFamides, Tachykinin-like peptide, and more (Pfeiffer and Homberg, 2014). Moreover, mapping metabotropic, G-protein-coupled receptors (GPCRs) of several neurotransmitters and neuromodulators to neurons of the CX show that chemical signaling and signal modulation are diverse and highly complex in the different compartments and circuits of the *Drosophila* CX (Kahsai et al., 2012). Only a few studies have addressed the role of these substances in locomotion. When the roles of two different neuropeptides (tachykinin and sNPF) in of Drosophila locomotor behavior, Kahsai et al. (2010) found that each is involved in different aspects of locomotion, orientation during locomotion, and locomotor activity levels, respectively. The initiation and maintenance of walking is context dependent and must be regulated by the internal state of the insect which depends on the metabolic and/or hormonal states and neuromodulatory systems. Such internal state must have an impact on the selection of a subpopulation of descending neurons dedicated to specific motor behavior such as, to name a few, walking, flight, grooming, righting.

Although the evolution of the brain in vertebrate and invertebrate phyla may follow a different “bauplan,” such difference does not imply that vertebrate and invertebrate motor systems are functionally different. Both systems control an articulated skeletal system using muscles which are in turn controlled by motor neurons. Notably, Strausfeld and Hirth (2013) draw functional similarities between the insect CX and the vertebrate basal ganglia as well as the insect GNG with the brain stem of the vertebrate brain. Future studies on different insects, combining electrophysiological, molecular, and behavioral studies with, for example, CRISPR-based genetics strategies might lead to answer these questions and further our understanding of the role of the head ganglia in walking and behavioral spontaneity. With this knowledge and by comparing the organization of locomotor system in invertebrates with that of vertebrates, we should uncover how such systems solve similar problems.

## Author Contributions

All authors contributed equally to the writing of this review.

## Conflict of Interest

The authors declare that the research was conducted in the absence of any commercial or financial relationships that could be construed as a potential conflict of interest.

## Abbreviations

CirC (connects the cerebral ganglia and the gnathal ganglia): circumesophageal connectives
CPGs: central pattern generators
CRG (formerly “supraesophageal ganglion” or brain): cerebral ganglia
CX: central complex
dFB: dorsal fan-shaped body
DINs: descending interneurons
EB (or central body lower: CBL): ellipsoid body
FB (or central body upper: CBU): fan-shaped body
GNG (formerly “subesophageal ganglion”): gnathal ganglia
LAL: lateral accessory lobe
MB: mushroom bodies
neck connectives: connects the gnathal ganglia (GNG) and the first (pro)thoracic ganglion
NO: paired noduli
PB: protocerebral bridge.

## References

[B1] Altman J. Kien J. (1987). “Functional organization of the subesophageal ganglion in arthropods,” in *Arthropod Brain: Its Evolution, Development, Structure and Function*, ed. Gupta A. P. (New York, NY: John Wiley & Sons), 265–301.

[B2] Arshavsky Y. I. Deliagina T. G. Orlovsky G. N. (1997). Pattern generation. *Curr. Opin. Neurobiol.* 7 781–789. 9464971 10.1016/s0959-4388(97)80136-5

[B3] Ayali A. Borgmann A. Bueschges A. Couzin-Fuchs E. Daun-Gruhn S. Holmes P. (2015). The comparative investigation of the stick insect and cockroach models in the study of insect locomotion. *Curr. Opin. Insect Sci.* 12 1–10.

[B4] Bässler U. Wegner U. (1983). Motor output of the denervated thoracic ventral nerve cord in the stick insect *Carausius morosus*. *J. Exp. Biol.* 105 127–145.

[B5] Bender J. A. Pollack A. J. Ritzmann R. E. (2010). Neural activity in the central complex of the insect brain is linked to locomotor changes. *Curr. Biol.* 20 921–926. 10.1016/j.cub.2010.03.054 20451382

[B6] Berkowitz A. Laurent G. (1996). Central generation of grooming motor patterns and interlimb coordination in locusts. *J. Neurosci.* 16 8079–8091. 8987833 10.1523/JNEUROSCI.16-24-08079.1996 PMC6579234

[B7] Bidaye S. S. Bockemühl T. Büschges A. (2017). Six-legged walking in insects: how CPGs, peripheral feedback, and descending signals generate coordinated and adaptive motor rhythms. *J. Neurophysiol.* 119 459–475. 10.1152/jn.00658.2017 29070634

[B8] Bidaye S. S. Machacek C. Wu Y. Dickson B. J. (2014). Neuronal control of *Drosophila* walking direction. *Science* 344 97–101. 24700860 10.1126/science.1249964

[B9] Böhm H. Schildberger K. (1992). Brain neurones involved in the control of walking in the cricket *Gryllus bimaculatus*. *J. Exp. Biol.* 166 113–130.

[B10] Borgmann A. Bueschges A. (2015). Insect motor control: methodological advances, descending control and inter-leg coordination on the move. *Curr. Opin. Neurobiol.* 33 8–15. 10.1016/j.conb.2014.12.010 25579064

[B11] Bräunig P. Burrows M. (2004). Projection patterns of posterior dorsal unpaired median neurons of the locust subesophageal ganglion. *J. Comp. Neurol.* 478 164–175. 15349977 10.1002/cne.20287

[B12] Burrows M. (1996). *The Neurobiology of an Insect Brain.* Oxford: Oxford University Press.

[B13] Cande J. Namiki S. Qiu J. Korff W. Card G. M. Shaevitz J. W. (2018). Optogenetic dissection of descending behavioral control in *Drosophila*. *Elife* 7:e34275. 10.7554/eLife.34275 PMC6031430 29943729

[B14] Combes D. Le Ray D. Lambert F. M. Simmers J. Straka H. (2008). An intrinsic feed-forward mechanism for vertebrate gaze stabilization. *Curr. Biol.* 18 R241–R243. 18364224 10.1016/j.cub.2008.02.018

[B15] Donlea J. M. Pimentel D. Miesenböck G. (2014). Neuronal machinery of sleep homeostasis in *Drosophila*. *Neuron* 81 860–872. 10.1016/j.neuron.2013.12.013 24559676 PMC3969244

[B16] Donlea J. M. Pimentel D. Talbot C. B. Kempf A. Omoto J. J. Hartenstein V. (2018). Recurrent circuitry for balancing sleep need and sleep. *Neuron* 97 378–389.e4. 10.1016/j.neuron.2017.12.016 29307711 PMC5779612

[B17] Donlea J. M. Thimgan M. S. Suzuki Y. Gottschalk L. Shaw P. J. (2011). Inducing sleep by remote control facilitates memory consolidation in *Drosophila*. *Science* 332 1571–1576. 10.1126/science.1202249 21700877 PMC4064462

[B18] Eaton R. C. Farley R. D. (1969). The neural control of cercal grooming behaviour in the cockroach, *Periplaneta americana*. *J. Insect Physiol.* 15 1047–1065. 5768887 10.1016/0022-1910(69)90143-7

[B19] Emanuel S. Libersat F. (2017). Do quiescence and wasp venom-induced lethargy share common neuronal mechanisms in cockroaches? *PloS One* 12:e0168032. 10.1371/journal.pone.0168032 28045911 PMC5207667

[B20] Gal R. Libersat F. (2006). New vistas on the initiation and maintenance of insect motor behaviors revealed by specific lesions of the head ganglia. *J. Comp. Physiol. A Neuroethol. Sens. Neural Behav. Physiol.* 192 1003–1020. 16733727 10.1007/s00359-006-0135-4

[B21] Graham D. (1979). Effects of circum-oesophageal lesion on the behaviour of the stick insect *Carausius morosus*. *Biol. Cybern.* 32 139–145.

[B22] Green J. Adachi A. Shah K. K. Hirokawa J. D. Magani P. S. Maimon G. (2017). A neural circuit architecture for angular integration in *Drosophila*. *Nature* 546 101–106. 10.1038/nature22343 28538731 PMC6320684

[B23] Green J. Vijayan V. Pires P. M. Adachi A. Maimon G. (2018). Walking *Drosophila* aim to maintain a neural heading estimate at an internal goal angle. *Biorxiv* [Preprint]. 10.1101/315796

[B24] Guo P. Ritzmann R. E. (2013). Neural activity in the central complex of the cockroach brain is linked to turning behaviors. *J. Exp. Biol.* 216 992–1002. 10.1242/jeb.080473 23197098

[B25] Harley C. Ritzmann R. (2010). Electrolytic lesions within central complex neuropils of the cockroach brain affect negotiation of barriers. *J. Exp. Biol.* 213 2851–2864. 10.1242/jeb.042499 20675555

[B26] Haspel G. Rosenberg L. A. Libersat F. (2003). Direct injection of venom by a predatory wasp into cockroach brain. *J. Neurobiol.* 56 287–292. 12884267 10.1002/neu.10238

[B27] Heinrich R. (2002). Impact of descending brain neurons on the control of stridulation, walking, and flight in orthoptera. *Microsc. Res. Tech.* 56 292–301. 11877804 10.1002/jemt.10033

[B28] Heisenberg M. (1994). Central brain function in insects: genetic studies on the mushroom bodies and central complex in *Drosophila*. *Fortschr. Zool.* 39 61–79.

[B29] Helfrich-Förster C. Wulf J. de Belle J. S. (2002). Mushroom body influence on locomotor activity and circadian rhythms in *Drosophila melanogaster*. *J. Neurogenet.* 16 73–109. 12479377 10.1080/01677060213158

[B30] Homberg U. (2015). Sky compass orientation in desert locusts—evidence from field and laboratory studies. *Front. Behav. Neurosci.* 9:346. 10.3389/fnbeh.2015.00346 26733834 PMC4679860

[B31] Honkanen A. Adden A. da Silva Freitas J. Heinze S. (2019). The insect central complex and the neural basis of navigational strategies. *J. Exp. Biol.* 222(Pt Suppl. 1):jeb188854. 10.1242/jeb.188854 30728235 PMC6474401

[B32] Hsu C. T. Bhandawat V. (2016). Organization of descending neurons in *Drosophila melanogaster*. *Sci. Rep.* 6:20259. 10.1038/srep20259 26837716 PMC4738306

[B33] Huber F. (1960). Untersuchungen über die Funktion des Zentralnervensystems und insbesondere des Gehirnes bei der Fortbewegung und der Lauterzeugung der Grillen. *Z. Vergl. Physiol.* 44 60–132.

[B34] Kahsai L. Carlsson M. A. Winther ÅM. Nässel D. R. (2012). Distribution of metabotropic receptors of serotonin, dopamine, GABA, glutamate, and short neuropeptide F in the central complex of *Drosophila*. *Neuroscience* 208 11–26. 10.1016/j.neuroscience.2012.02.007 22361394

[B35] Kahsai L. Martin J.-R. Winther ÅM. (2010). Neuropeptides in the *Drosophila* central complex in modulation of locomotor behavior. *J. Exp. Biol.* 213 2256–2265. 20543124 10.1242/jeb.043190

[B36] Kaiser M. Arvidson R. Zarivach R. Adams M. E. Libersat F. (2019). Molecular cross-talk in a unique parasitoid manipulation strategy. *Insect Biochem. Mol. Biol.* 106 64–78. 30508629 10.1016/j.ibmb.2018.11.009

[B37] Kaiser M. Libersat F. (2015). The role of the cerebral ganglia in the venom-induced behavioral manipulation of cockroaches stung by the parasitoid jewel wasp. *J. Exp. Biol.* 218 1022–1027. 25687435 10.1242/jeb.116491

[B38] Kien J. (1983). The initiation and maintenance of walking in the locust: an alternative to the command concept. *Proc. R. Soc. Lond. B Biol. Sci.* 219 137–174.

[B39] Kien J. (1990a). Neuronal activity during spontaneous walking– I. Starting and stopping. *Comp. Biochem. Physiol. A Comp. Physiol.* 95 607–621. 1971547 10.1016/0300-9629(90)90747-g

[B40] Kien J. (1990b). Neuronal activity during spontaneous walking– II. Correlation with stepping. *Comp. Biochem. Physiol. A Comp. Physiol.* 95 623–638. 1971548 10.1016/0300-9629(90)90748-h

[B41] Kien J. Fletcher W. Altman J. Ramirez J.-M. Roth U. (1990). Organisation of intersegmental interneurons in the suboesophageal ganglion of *Schistocerca gregaria* (Forksal) and *Locusta migratoria* migratorioides (Reiche & Fairmaire) (Acrididae, Orthoptera). *Int. J. Insect Morphol. Embryol.* 19 35–60.

[B42] Knebel D. Ayali A. Pflueger H.-J. Rillich J. (2017). Rigidity and flexibility: the central basis of inter-leg coordination in the locust. *Front. Neural Circuits* 10:112. 10.3389/fncir.2016.00112 28123358 PMC5225121

[B43] Knebel D. Rillich J. Nadler L. Pflueger H.-J. Ayali A. (2019). The functional connectivity between the locust leg pattern generators and the subesophageal ganglion higher motor center. *Neurosci. Lett.* 692 77–82. 30391322 10.1016/j.neulet.2018.10.060

[B44] Knop G. Denzer L. Büschges A. (2001). A central pattern-generating network contributes to “Reflex-Reversal”–like leg motoneuron activity in the locust. *J. Neurophysiol.* 86 3065–3068. 11731562 10.1152/jn.2001.86.6.3065

[B45] Kononenko N. L. Hartfil S. Willer J. Ferch J. Wolfenberg H. Pflueger H. J. (2019). A population of descending tyraminergic/octopaminergic projection neurons of the insect deutocerebrum. *J. Comp. Neurol.* 527 1027–1038. 10.1002/cne.24583 30444529

[B46] Libersat F. Pflueger H.-J. (2004). Monoamines and the orchestration of behavior. *Bioscience* 54 17–25.

[B47] Liu Q. Liu S. Kodama L. Driscoll M. R. Wu M. N. (2012). Two dopaminergic neurons signal to the dorsal fan-shaped body to promote wakefulness in *Drosophila*. *Curr. Biol.* 22 2114–2123. 10.1016/j.cub.2012.09.008 23022067 PMC3505250

[B48] Liu S. Liu Q. Tabuchi M. Wu M. N. (2016). Sleep drive is encoded by neural plastic changes in a dedicated circuit. *Cell* 165 1347–1360. 10.1016/j.cell.2016.04.013 27212237 PMC4892967

[B49] Mabuchi I. Shimada N. Sato S. Ienaga K. Sakai T. (2016). Mushroom body signaling is required for locomotor activity rhythms in *Drosophila*. *Neurosci. Res.* 111 25–33. 27106579 10.1016/j.neures.2016.04.005

[B50] Marder E. Bucher D. (2001). Central pattern generators and the control of rhythmic movements. *Curr. Biol.* 11 R986–R996. 11728329 10.1016/s0960-9822(01)00581-4

[B51] Marder E. Bucher D. Schulz D. J. Taylor A. L. (2005). Invertebrate central pattern generation moves along. *Curr. Biol.* 15 R685–R699. 16139202 10.1016/j.cub.2005.08.022

[B52] Martin J. P. Guo P. Mu L. Harley C. M. Ritzmann R. E. (2015). Central-complex control of movement in the freely walking cockroach. *Curr. Biol.* 25 2795–2803. 26592340 10.1016/j.cub.2015.09.044

[B53] Martin J.-R. Ernst R. Heisenberg M. (1998). Mushroom bodies suppress locomotor activity in *Drosophila melanogaster*. *Learn. Mem.* 5 179–191. 10454382 PMC311252

[B54] Martin J.-R. Raabe T. Heisenberg M. (1999). Central complex substructures are required for the maintenance of locomotor activity in *Drosophila melanogaster*. *J. Comp. Physiol. A* 185 277–288. 10573866 10.1007/s003590050387

[B55] Mizunami M. Iwasaki M. Okada R. Nishikawa M. (1998a). Topography of four classes of Kenyon cells in the mushroom bodies of the cockroach. *J. Comp. Neurol.* 399 162–175. 9721901 10.1002/(sici)1096-9861(19980921)399:2<162::aid-cne2>3.0.co;2-z

[B56] Mizunami M. Iwasaki M. Okada R. Nishikawa M. (1998b). Topography of modular subunits in the mushroom bodies of the cockroach. *J. Comp. Neurol.* 399 153–161. 9721900 10.1002/(sici)1096-9861(19980921)399:2<153::aid-cne1>3.0.co;2-#

[B57] Mizunami M. Okada R. Li Y. Strausfeld N. J. (1998c). Mushroom bodies of the cockroach: activity and identities of neurons recorded in freely moving animals. *J. Comp. Neurol.* 402 501–519. 9862323

[B58] Namiki S. Dickinson M. H. Wong A. M. Korff W. Card G. M. (2018). The functional organization of descending sensory-motor pathways in *Drosophila*. *Elife* 7:e34272. 10.7554/eLife.34272 29943730 PMC6019073

[B59] Namiki S. Kanzaki R. (2016). Comparative neuroanatomy of the lateral accessory lobe in the insect brain. *Front. Physiol.* 7:244. 10.3389/fphys.2016.00244 27445837 PMC4917559

[B60] Okada R. Sakura M. Mizunami M. (2003). Distribution of dendrites of descending neurons and its implications for the basic organization of the cockroach brain. *J. Comp. Neurol.* 458 158–174. 12596256 10.1002/cne.10580

[B61] Pfeiffer K. Homberg U. (2014). Organization and functional roles of the central complex in the insect brain. *Annu. Rev. Entomol.* 59 165–184. 24160424 10.1146/annurev-ento-011613-162031

[B62] Pimentel D. Donlea J. M. Talbot C. B. Song S. M. Thurston A. J. Miesenböck G. (2016). Operation of a homeostatic sleep switch. *Nature* 536 333–337. 10.1038/nature19055 27487216 PMC4998959

[B63] Poulet J. F. Hedwig B. (2006). The cellular basis of a corollary discharge. *Science* 311 518–522. 16439660 10.1126/science.1120847

[B64] Reingold S. C. Camhi J. M. (1977). A quantitative analysis of rhythmic leg movements during three different behaviors in the cockroach, *Periplaneta americana*. *J. Insect Physiol.* 23 1407–1420.

[B65] Ridgel A. L. Ritzmann R. E. (2005). Effects of neck and circumoesophageal connective lesions on posture and locomotion in the cockroach. *J. Comp. Physiol. A Neuroethol. Sens. Neural Behav. Physiol.* 191 559–573. 15864596 10.1007/s00359-005-0621-0

[B66] Rillich J. Stevenson P. A. Pflueger H. J. (2013). Flight and walking in locusts–cholinergic co-activation, temporal coupling and its modulation by biogenic amines. *PloS One* 8:e62899. 10.1371/journal.pone.0062899 23671643 PMC3650027

[B67] Ritzmann R. E. Harley C. M. Daltorio K. A. Tietz B. R. Pollack A. J. Bender J. A. (2012). Deciding which way to go: how do insects alter movements to negotiate barriers? *Front. Neurosci.* 6:97. 10.3389/fnins.2012.00097 22783160 PMC3390555

[B68] Ritzmann R. E. Quinn R. D. (2004). “Locomotion in complex terrain,” in *Walking: Biological and Technological Aspects. International Centre for Mechanical Sciences (Courses and Lectures)*, eds Pfeiffer F. Zielinska T. (Vienna: Springer).

[B69] Ritzmann R. E. Zill S. N. (2017). “Control of locomotion in hexapods,” in *The Oxford Handbook of Invertebrate Neurobiology*, ed. Byrne J. H. (Oxford: Oxford University Press), 1–28.

[B70] Roeder K. D. (1937). The control of tonus and locomotor activity in the praying mantis (*Mantis religiosa* L.). *J. Exp. Zool.* 76 353–374.

[B71] Rosenberg L. Pflueger H. Wegener G. Libersat F. (2006). Wasp venom injected into the prey’s brain modulates thoracic identified monoaminergic neurons. *J. Neurobiol.* 66 155–168. 16215998 10.1002/neu.20203

[B72] Ryckebusch S. Laurent G. (1993). Rhythmic patterns evoked in locust leg motor neurons by the muscarinic agonist pilocarpine. *J. Neurophysiol.* 69 1583–1595. 8389831 10.1152/jn.1993.69.5.1583

[B73] Seelig J. D. Jayaraman V. (2015). Neural dynamics for landmark orientation and angular path integration. *Nature* 521 186–191. 10.1038/nature14446 25971509 PMC4704792

[B74] Selverston A. I. (2010). Invertebrate central pattern generator circuits. *Philos. Trans. R. Soc. B Biol. Sci.* 365 2329–2345. 10.1098/rstb.2009.0270 PMC2894947 20603355

[B75] Serway C. N. Kaufman R. R. Serway C. N. Kaufman R. R. Strauss R. de Belle J. S. (2009). Mushroom bodies enhance initial motor activity in *Drosophila*. *J. Neurogenet.* 23 173–184. 10.1080/01677060802572895 19145515

[B76] Sombati S. Hoyle G. (1984). Generation of specific behaviors in a locust by local release into neuropil of the natural neuromodulator octopamine. *J. Neurobiol.* 15 481–506. 6097645 10.1002/neu.480150607

[B77] Staudacher E. (1998). Distribution and morphology of descending brain neurons in the cricket *Gryllus bimaculatus*. *Cell Tissue Res.* 294 187–202. 9724469 10.1007/s004410051169

[B78] Strausfeld N. J. Hirth F. (2013). Deep homology of arthropod central complex and vertebrate basal ganglia. *Science* 340 157–161. 10.1126/science.1231828 23580521

[B79] Strauss R. (2002). The central complex and the genetic dissection of locomotor behaviour. *Curr. Opin. Neurobiol.* 12 633–638. 12490252 10.1016/s0959-4388(02)00385-9

[B80] Strauss R. Heisenberg M. (1993). A higher control center of locomotor behavior in the *Drosophila* brain. *J. Neurosci.* 13 1852–1861. 8478679 10.1523/JNEUROSCI.13-05-01852.1993 PMC6576564

[B81] Tschida K. Bhandawat V. (2015). Activity in descending dopaminergic neurons represents but is not required for leg movements in the fruit fly *Drosophila*. *Physiol. Rep.* 3:e12322. 10.14814/phy2.12322 25742959 PMC4393157

[B82] Turner-Evans D. Wegener S. Rouault H. Franconville R. Wolff T. Seelig J. D. (2017). Angular velocity integration in a fly heading circuit. *Elife* 6:e23496. 10.7554/eLife.23496 28530551 PMC5440168

[B83] Turner-Evans D. B. Jayaraman V. (2016). The insect central complex. *Curr. Biol.* 26 R453–R457. 10.1016/j.cub.2016.04.006 27269718

[B84] Varga A. G. Ritzmann R. E. (2016). Cellular basis of head direction and contextual cues in the insect brain. *Curr. Biol.* 26 1816–1828. 10.1016/j.cub.2016.05.037 27397888

[B85] Wilson D. M. (1961). The central nervous control of flight in a locust. *J. Exp. Biol.* 38 471–490.

[B86] Wolff T. Iyer N. A. Rubin G. M. (2015). Neuroarchitecture and neuroanatomy of the *Drosophila* central complex: a GAL4−based dissection of protocerebral bridge neurons and circuits. *J. Comp. Neurol.* 523 997–1037. 25380328 10.1002/cne.23705 PMC4407839

[B87] Wosnitza A. Bockemühl T. Dübbert M. Scholz H. Büschges A. (2013). Inter-leg coordination in the control of walking speed in *Drosophila*. *J. Exp. Biol.* 216 480–491. 10.1242/jeb.078139 23038731

[B88] Yellman C. Tao H. He B. Hirsh J. (1997). Conserved and sexually dimorphic behavioral responses to biogenic amines in decapitated *Drosophila*. *Proc. Natl. Acad. Sci. U.S.A.* 94 4131–4136. 9108117 10.1073/pnas.94.8.4131 PMC20580

[B89] Zacarias R. Namiki S. Card G. M. Vasconcelos M. L. Moita M. A. (2018). Speed dependent descending control of freezing behavior in *Drosophila melanogaster*. *Nat. Commun.* 9:3697. 10.1038/s41467-018-05875-1 30209268 PMC6135764

[B90] Zill S. Schmitz J. Büschges A. (2004). Load sensing and control of posture and locomotion. *Arthropod Struct. Dev.* 33 273–286. 18089039 10.1016/j.asd.2004.05.005

[B91] Zorović M. Hedwig B. (2013). Descending brain neurons in the cricket *Gryllus bimaculatus* (de Geer): auditory responses and impact on walking. *J. Comp. Physiol. A Neuroethol. Sens. Neural Behav. Physiol.* 199 25–34. 23104703 10.1007/s00359-012-0765-7

